# Coral assemblages at higher latitudes favor short‐term potential over long‐term performance

**DOI:** 10.1002/ecy.4138

**Published:** 2023-07-30

**Authors:** James Cant, James D. Reimer, Brigitte Sommer, Katie M. Cook, Sun W. Kim, Carrie A. Sims, Takuma Mezaki, Cliodhna O'Flaherty, Maxime Brooks, Hamish A. Malcolm, John M. Pandolfi, Roberto Salguero‐Gómez, Maria Beger

**Affiliations:** ^1^ Centre for Biological Diversity University of St Andrews St Andrews UK; ^2^ School of Biology, Faculty of Biological Sciences University of Leeds Leeds UK; ^3^ Molecular Invertebrate Systematics and Ecology Laboratory Graduate School of Engineering and Science, University of the Ryukyus Nishihara Japan; ^4^ Tropical Biosphere Research Centre University of the Ryukyus Nishihara Japan; ^5^ School of Life and Environmental Science The University of Sydney Camperdown New South Wales Australia; ^6^ School of Life Sciences University of Technology Sydney Ultimo New South Wales Australia; ^7^ National Institute of Water and Atmospheric Research Hamilton New Zealand; ^8^ Australian Research Council Centre of Excellence for Coral Reef Studies, School of Biological Sciences The University of Queensland Brisbane Queensland Australia; ^9^ Smithsonian Tropical Research Institute Panama City Republic of Panama; ^10^ Kuroshio Biological Research Foundation, Nishidomari, Otsuki‐cho Kochi Japan; ^11^ Fisheries Research, Department of Primary Industries Coffs Harbour New South Wales Australia; ^12^ Department of Zoology University of Oxford Oxford UK; ^13^ Centre for Biodiversity and Conservation Science, School of Biological Sciences University of Queensland Brisbane Queensland Australia; ^14^ Max Planck Institute for Demographic Research Rostock Germany

**Keywords:** amplification, demography, integral projection model (IPM), resilience, subtropical, transient dynamics

## Abstract

The persistent exposure of coral assemblages to more variable abiotic regimes is assumed to augment their resilience to future climatic variability. Yet, while the determinants of coral population resilience across species remain unknown, we are unable to predict the winners and losers across reef ecosystems exposed to increasingly variable conditions. Using annual surveys of 3171 coral individuals across Australia and Japan (2016–2019), we explore spatial variation across the short‐ and long‐term dynamics of competitive, stress‐tolerant, and weedy assemblages to evaluate how abiotic variability mediates the structural composition of coral assemblages. We illustrate how, by promoting short‐term potential over long‐term performance, coral assemblages can reduce their vulnerability to stochastic environments. However, compared to stress‐tolerant, and weedy assemblages, competitive coral taxa display a reduced capacity for elevating their short‐term potential. Accordingly, future climatic shifts threaten the structural complexity of coral assemblages in variable environments, emulating the degradation expected across global tropical reefs.

## INTRODUCTION

Anticipating the resilience of natural communities requires an in‐depth understanding for the determinants underpinning their constituent populations' responses to recurrent disturbances (Vázquez et al., 2017; Williams et al., 2008). Changes in environmental regimes provoke spatial shifts in the performance and distribution of populations, which upscale to the compositional reassembly of biological communities (Pecl et al., 2017; Totland & Nyléhn, 1998). Exposure to more variable environments is expected to indirectly augment community resilience (Boyd et al., 2016; Rivest et al., 2017). However, nuanced relationships between population characteristics and biophysical conditions ensure inconsistent responses to climate shifts (Parmesan & Yohe, 2003); with differential population sensitivities to habitat change having both accelerated and reversed expected poleward range shifts in response to climate warming (Chen et al., 2011). By linking the mechanisms driving heterospecific variation across population responses to environmental change, one can predict the resilience of whole communities to increased climatic variability (Dawson et al., 2011; Foden et al., 2013; Williams et al., 2008).

Located at the interface between tropical and temperate ecoregions, subtropical coral assemblages provide an opportunity for exploring the determinants of population resilience (Beger et al., 2014; Burt et al., 2020; Camp et al., 2018). Recently, subtropical coral assemblages have undergone transformation, with tropical coral taxa undergoing poleward range expansions in response to shifting thermal regimes (Baird et al., 2012; Booth & Sears, 2018; Precht & Aronson, 2004; Tuckett et al., 2017; Yamano et al., 2011). At higher latitudes, however, coral assemblages are exposed to enhanced seasonality and cooler temperatures and, thus, experience greater abiotic variability relative to their tropical counterparts (Sommer et al., 2018). Subsequently, corals in subtropical regions offer insight into how differing coral populations utilize strategies to mediate their performance in response to environmental stochasticity across community‐ and regional‐scales.

Exploring the performance of populations exposed to environmental stochasticity requires a consideration of their transient (i.e., short‐term) dynamics (Cant, Cook, et al., 2022; Ezard et al., 2010; Hastings, 2004; Hastings et al., 2018). Asymptotic (i.e., long‐term) population growth rate (λ), which describes temporal changes in population size at stationary equilibrium (Caswell, 2001), is the predominant metric used to quantify population performance (Caswell, 2001; Crone et al., 2011). However, stochastic conditions maintain natural populations within a transient state, preventing the emergence of stationary equilibria (Hastings, 2001, 2004; Hastings et al., 2018). Within stochastic environments, recurrent disturbances impose short‐term changes upon the structure of populations that can elevate (i.e., demographic amplification) or diminish (i.e., demographic attenuation) their growth rates, resulting in population performance characteristics deviating from long‐term expectations (Ezard et al., 2010; Stott et al., 2011). Quantifying how transient population performance (henceforth short‐term potential) deviates from long‐term expectations therefore is crucial for predicting the success or failure of natural populations (Koons et al., 2005), an approach that remains neglected within coral research (Cant, Salguero‐Gómez, & Beger, 2022).

In species rich communities, evaluating ecological dynamics requires a trait‐based approach to condense vast quantities of demographic detail (Chalmandrier et al., 2021). Given the diversity of coral assemblages, exploring patterns across the demographic characteristics of co‐occurring species presents a logistical challenge (Madin, Anderson, et al., 2016). Yet, this is a challenge that can be navigated by pooling individuals based on shared trait characteristics. Morphological, physiological, and phenological functional traits influence the fitness of individuals and thus determine the demographic characteristics of their populations (Violle et al., 2007), their responses to disturbances (Grime & Pierce, 2012), and subsequently the assembly of biological communities (Cadotte et al., 2011; Falster et al., 2017; McGill et al., 2006). Indeed, functional trait characteristics impact upon the demographic properties of coral populations (e.g., colony growth and reproduction [Álvarez‐Noriega et al., 2016; Madin et al., 2012]), mediating their ability to respond to local abiotic patterns (Sommer et al., 2014). Given such strong links between coral traits and demographic performance, the categorization of coral taxa into competitive, stress tolerant, generalist, and weedy life‐history assemblages (sensu Darling et al., 2012) can be used to evaluate broadscale patterns in coral community reassembly (Darling et al., 2013, 2019; Zinke et al., 2018). Trait‐based assessments of coral community assembly also better inform upon the wider implications of ongoing community shifts than taxonomic‐based assessments, thereby aiding the management of coral reef ecosystems (Darling et al., 2019).

**FIGURE 1:**
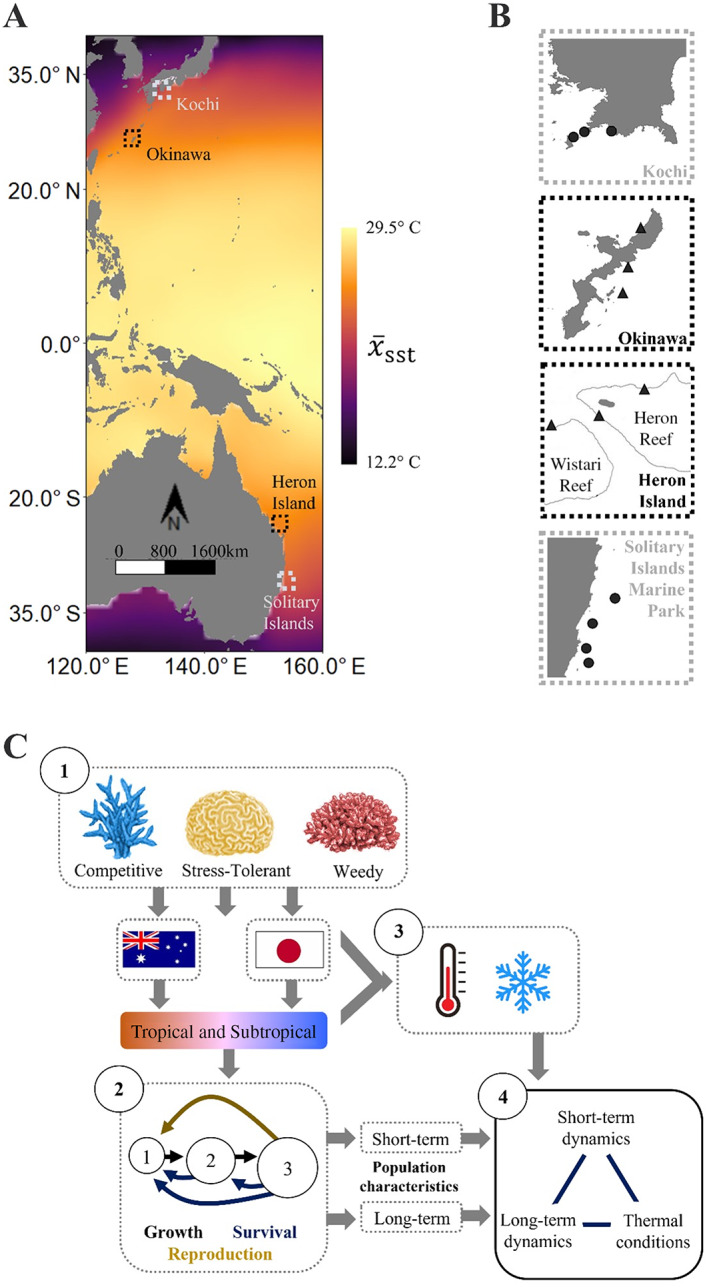
Using repeated annual surveys of tagged individual colonies, conducted between 2016 and 2019, we quantified the influence of environmental stochasticity on the long‐term performance and short‐term potential of tropical and subtropical coral populations in southern Japan and eastern Australia. (A) As climate shifts induce range expansions in many coral species worldwide, their populations are increasingly exposed to a gradient in thermal regimes, illustrated here by mean monthly sea surface temperatures (*x̄*_sst_; degree Celsius; sst, sea surface temperature) recorded between 1950 and 2019 (Rayner et al., 2003). (B) Between 2016 and 2019, we documented the survival, growth, fragmentation, and recruitment patterns of 3171 tagged coral individuals within the tropical reef communities (▲) of Okinawa (Japan) and Heron Island (Australia), and within the subtropical communities (●) of Kochi (Japan) and the Solitary Islands Marine Park (Australia). (C) Using these data, we parameterized integral projection models (IPMs) describing the dynamics of tropical and subtropical assemblages of competitive, stress‐tolerant, and weedy coral taxa. Combining outputs obtained from these models with measures of the thermal regimes experienced by each population, we then explored the relationships between the long‐term performance and short‐term potential of coral populations, and their exposure to gradients in abiotic variability.

Here, we investigate how the demographic characteristics of competitive, stress‐tolerant, and weedy coral assemblages map onto patterns of abiotic variability associated with the transition between tropical and subtropical environments. Exploiting gradients across the differing dimensions of thermal variability (monthly mean sea surface temperature [SST], monthly SST variance, and monthly SST frequency spectrum) as a key measure of abiotic variability (McIlroy et al., 2019; Toth et al., 2021), we used integral projection models (IPMs; Easterling et al., 2000) to quantify the association between abiotic variability and the short‐term potential and long‐term performance characteristics of tropical and subtropical coral assemblages across both the northern and southern hemispheres (Figure 1). Describing how state‐specific patterns in individual survival, development, and reproduction translate into population‐level characteristics, IPMs offer an approach for quantifying how abiotic environments influence population viability (Merow et al., 2014). Specifically, we anticipate that, compared to their tropical counterparts, subtropical coral assemblages will prioritize short‐term potential over long‐term performance, corresponding with the need for subtropical coral populations to endure periodically disturbed environments. Thus, we expect that characteristics associated with enhanced short‐term potential will align with more variable abiotic conditions, whereas measures of long‐term performance will be greater in more consistent environments; a pattern that will persist irrespective of functional strategy and geographic location.

## METHODS

### Modeling population dynamics

IPMs capture how the state composition of individuals influences the performance of populations over discrete time periods (*t* to *t* + 1; Easterling et al., 2000). IPMs build upon the well‐established framework of Matrix Population Models, which are used to project population characteristics using a discrete matrix categorizing the survival, developmental, and reproductive rates of individuals depending on their position along a series of discrete state classes (e.g., age or developmental stage; Rees et al., 2014). However, an IPM offers greater flexibility, as it allows for quantifying the performance of populations in which the state of individuals is characterized along a continuous scale (e.g., size; Easterling et al., 2000). Here, to quantify the long‐term performance characteristics and short‐term potential of coral populations, we used IPMs describing patterns in colony survival (σ), transitions in size (growth and shrinkage, γ), fragmentation probability (κ), fecundity (φ), and recruitment (ϕ), each as a function of colony size (*z*; visible horizontal surface area, square centimeters). Specifically, our IPMs took the form

(1) $$n\left[z^{\prime },t+1\right]=\int_{L}^{U}\left(P_{z^{\prime }z}+F_{z^{\prime }z}\;n\left[z,t\right]\;\delta z\right),$$

(2) $$P_{z^{\prime }z}=\left(1-\kappa_{z}\right)\sigma_{z}\;\gamma_{z^{\prime }z}+\left(\;\kappa_{z}\;\kappa b_{z}\;\kappa_{z}^{0}\right),$$

(3) $$F_{z^{\prime }z}=\varphi_{z}\;\phi C_{0},$$

with [*L*, *U*] representing the range of possible colony sizes; calculated as 10% above and below observed maximum and minimum colony sizes to avoid accidental exclusion (Williams et al., 2012). Accordingly, the structure of a population at time *t* + 1 (*n*[*z*′, *t* + 1]) is a product of its structure at time *t* (*n*[*z*′, *t*]) subject to the survival (σ_*z*_) and transition of individual colonies from size *z* to size *z*′ (γ_*z*′*z*_); the probability of colony fragmentation (κ_*z*_) and the number (κ*b*_*z*_) and size distribution of any colony remnants produced ($\kappa_{z}^{0}$); and colony fecundity (φ_*z*_) combined with the probability of successful recruitment (ϕ) and the size distribution of surviving recruits (C_0_).

### Data collection

We parameterized our IPMs using data collected during annual surveys of 3171 tagged colonies within tropical and subtropical coral assemblages in southern Japan and eastern Australia (Figure 1). We tagged individual colonies using permanent plots arranged haphazardly throughout four focal coral assemblages (Australian subtropics [AS], Australian tropics [AT], Japanese subtropics [JS], Japanese tropics [JT]) and demarcated with numbered tags (Cant et al., 2021; Cant, Cook, et al., 2022). Repeated surveys of these four assemblages were carried out between 2016 and 2019, although survey length was not equal across each assemblage with, (1) the date of initial colony tagging differing across each region (AS = 2016, AT = 2018, JS and JT = 2017; Appendix S1: Section S1), and (2) the coronavirus (COVID‐19) pandemic preventing further surveys in 2020. All tagged colonies were identified either in situ or from photographs to the lowest possible taxonomic level (either genus or species). No samples were taken from tagged colonies, as although this would have allowed us to resolve species identity, we wanted to avoid any lasting interference with the processes of colony survival, growth, and fragmentation.

To facilitate comparing population characteristics observed across spatially distinct regions in Australia and Japan with varying degrees of species overlap (Veron et al., 2016), we grouped tagged colonies across each region according to shared life‐history strategies (sensu Darling et al., 2012, 2013; Zinke et al., 2018). Specifically, we categorized colonies as “competitive,” “weedy,” “stress‐tolerant” or “generalist” based on their morphology, growth rate and reproductive mode, following the genera classifications of Darling et al. (2012), with minor adaptions made based on local expertise (see Appendix S1: Section S2 for a detailed list). In the event that genera represented species classified across multiple categories (19 cases), we randomly assigned individuals across the relevant categories in proportion with the number of species within each category known to occur in the area (sensu Zinke et al., 2018). Following the pooling of colonies according to their life‐history strategies, we omitted all individuals defined as generalists from subsequent analyses due to their limited representation across our regional samples (*n*: AS = 22 colonies, AT = 31, JS = 17, JT = 65). Consequently, we constructed IPMs concerning the dynamics of each functional coral assemblage (competitive, stress‐tolerant, and weedy; Figure 1) at each of the four geographical locations.

Photographs capturing the visible horizontal extent of tagged colonies were used to follow individuals over successive surveys and obtain longitudinal records of colony surface area (square centimeters; transformed to a log scale) over time. Using generalized linear mixed models (GLMMs), we estimated size‐specific patterns in colony survival (σ), transitions in size (γ), and fragmentation probability (κ) for each population (Appendix S1: Section S1). In each case, our GLMMs included random effects (colony identity and survey location) to account for any autocorrelation between observations and within‐subject variability associated with our pooling of data recorded from individuals followed across multiple years, and at different sites. Colony survival (σ) reflected the continued presence of tagged individuals across survey intervals (*t* to *t* + 1) and was modeled as a logistic function of colony surface area at time *t*. Colony size transitions (γ), representing both growth through colony extension, and shrinkage through partial mortality (Madin et al., 2020), were modeled using the polynomial relationship between initial colony surface area at time *t* and subsequent surface area at time *t* + 1. Colony fragmentation probability (κ) was then modeled as a polynomial logistic function of colony size at time *t*. During our surveys, we recorded fragmentation in the event of observed colony breakage, recording the size (surface area, square centimeters) of all remnants produced in each case. Subsequently, we also modeled the number (κ*b*_*z*_) and size ($\kappa_{z}^{0}$) of remnant colonies produced during fragmentation as a function of colony size at time *t*, using Poisson and polynomial GLMMs, respectively.

Alongside our surveys of tagged individual colonies, we also monitored colony recruitment within our permanent coral plots. During each annual survey, we recorded the number and size of all new corals <5 cm diameter appearing within each plot (encompassing both new recruits and colonies that were undetectable during previous years) to quantify annual and regional variability in the density of corals newly establishing/recruiting into our plots (Appendix S1: Table S2), as well as estimate population‐specific new colony size distributions (C_0_; Appendix S1: Section S1). Prior to incorporating recruitment dynamics into our IPMs, however, we first determined patterns in colony fecundity (φ). Using data relating colony size and larval output (larval density, cubic centimeters) extracted from the Coral Trait Database (Hall & Hughes, 1996; Madin, Hoogenboom, et al., 2016), we calculated colony fecundity (φ) as the polynomial relationship between colony size at *t* and expected larval output (Appendix S1: Section S1). This approach enabled us to complete the life cycle loop, linking the dynamics of existing individuals with the introduction of new, genetically distinct individuals within our IPMs; a necessary step when evaluating population performance (Caswell, 2001). However, to ensure our modeled recruitment dynamics, and therefore, our modeling framework, reflected empirical observations, we parameterized a new colony settlement function (ϕ) into our IPMs. As a probability‐based function, this new colony settlement function converts modeled larval outputs into proportional newly establishing colony densities corresponding with our empirical counts. We determined this new colony settlement function by dividing total expected larval output in any given year by the corresponding annual new colony count (Appendix S1: Section S1, sensu Bramanti et al., 2015; Cant et al., 2021).

### Quantifying population characteristics

From our IPMs, we obtained estimates of long‐term performance (asymptotic population growth, λ), generation time (*T*), and short‐term potential (damping ratio [ρ], maximal amplification [${\bar{\rho }}_{\max}$] and transient envelope [TE]) for each tropical and subtropical coral assemblage (Capdevila et al., 2020; Caswell, 2001; Gaillard et al., 2005; Stott et al., 2010, 2011). Estimates of λ are typically used as a measure of long‐term population viability (Crone et al., 2011), and reflect whether a population is expected to grow (λ > 1) or decline (λ < 1) when at stationary equilibrium (Caswell, 2001). Generation time is a measure of population turnover, describing the time needed for individuals of a population to be replaced (Gaillard et al., 2005). Alternatively, our measures of short‐term potential describe the expected characteristics of populations following their displacement from stationary equilibrium due to disturbances. The damping ratio constitutes a measure of demographic recovery (Capdevila et al., 2020; Hodgson et al., 2015), describing the rate at which a population perturbed from its stationary equilibrium converges back to its asymptotic growth trajectory (Caswell, 2001). Meanwhile, maximal amplification quantifies the greatest increase in population size following a disturbance, relative to its asymptotic growth trajectory (Stott et al., 2010, 2011). Finally, the TE quantifies the magnitude by which the short‐term dynamics of a population deviate from its long‐term trajectory (Capdevila et al., 2020).

To calculate the aforementioned demographic characteristics, we discretized our IPMs into large matrices. Applying the “midpoint rule,” we integrated each IPM into a high‐dimension matrix (200 × 200 cells), with the probability of transitioning from one cell to the next approximated at the cell midpoint and multiplied by the cell width as per Zuidema et al. (2010). Estimates of λ were then identified as the dominant eigenvalue of each discretized matrix, while we estimated damping ratios as the ratio between each matrice subdominant and dominant eigenvalues. With the R package Rage (Jones et al., 2021), we then calculated generation time using estimates of net reproductive rate (*R*_0_) and λ obtained from each matrix,

(4) $$T=\frac{\log\left(R_{0}\right)}{\log\left(\lambda \right)}.$$

Next, we determined the TE of each assemblage using their associated Kreiss bounds of amplification (${\bar{K}}_{\lambda }^{*}$) and attenuation (${\underline{K}}_{\lambda }^{*}$),

(5) $$\mathrm{TE}={\bar{K}}_{\lambda }^{*}-{\underline{K}}_{\lambda }^{*}.$$

Respectively, the Kreiss bounds of amplification and attenuation reflect the largest and smallest expected long‐term densities of a population following the dissipation of transient conditions, relative to its asymptotic growth trajectory (Kreiss, 1962; Townley et al., 2007; Townley & Hodgson, 2008). We acknowledge here that this definition is more commonly applied to measures of population inertia (Stott et al., 2011), which are more typically used in estimating TEs (Capdevila et al., 2020). However, Kreiss bound estimates have been demonstrated to align with corresponding estimates of population inertia and, unlike estimates of population inertia, are not sensitive to imprimitive population models (i.e., non‐negative models permitting transitions between all state classes, but with transitions between certain stages occurring only at periodic intervals [Caswell, 2001; Stott et al., 2011]), hence their selection here. We derived these Kreiss bounds, alongside estimates of maximal amplification, using their corresponding functions in the R package popdemo (Stott et al., 2012).

Across each demographic measure, we determined the variance in our assemblage‐specific estimates through Jack‐knife resampling. During resampling, we generated 1000 IPM variants for each assemblage, each time using 95% of our original data sample without replacement, while permitting recruit survival probabilities (ϕ) to vary within observed limits. Finally, prior to their inclusion in further analyses, the jack‐knifed distributions of λ, generation time, TE, and maximal amplification each required transforming to ensure approximate normality. We omitted 26 variants for which λ > 2, as these presented unrealistic illustrations of population performance (i.e., more than doubling population size every year), before applying a log transformation to the generation time variable and a power transformation (*y* ^*x*^) across the damping ratio (*y* ^−2.0^), TE (*y* ^−0.1^), and maximal amplification variables (*y* ^−0.5^).

### Evaluating spatial trends in population characteristics

To test for patterns in the spatial variation of long‐term performance and short‐term potential across tropical and subtropical coral assemblages, we utilized partial least squares regression (PLSR), analysis of variance (ANOVA), and Type 2 linear regression. Initially, we applied PLSR to test whether contrasting patterns in the long‐term performance characteristics and short‐term potential of coral assemblages align with their exposure to abiotic variability. A PLSR regression quantifies the association between multiple predictor variables and one or more dependant variables (Carrascal et al., 2009). Subsequently, using this technique we simultaneously evaluated the relationships between mean estimates of λ, damping ratio, and TE obtained for each assemblage, and their correlation with patterns in abiotic variability, to provide an insight into the demographic trade‐offs of coral assemblages and their mechanistic drivers.

To evaluate how abiotic variability mediates the selection of short‐ and long‐term performance characteristics in coral assemblages, within our PLSR analyses, we represented the abiotic variability experienced by each coral assemblage using three measures of local SST variability: mean monthly SST (*x̄*_sst_), monthly SST variance (cv_sst_), and monthly SST frequency spectrum (β_sst_; Appendix S1: Section S3). Focusing on the four geographical regions in which our focal coral assemblages were surveyed (GPS: AS = −30.3°, 153.1°; AT = −23.4°, 151.9°; JS = 32.8°, 132.6°; JT = 26.5°, 128.1°; Figure 1), we extracted monthly SST readings (degree Celsius; overlaid on a 1° latitude‐longitude grid) taken between January 1950 and December 2019, inclusive, from the HadISST dataset (Rayner et al., 2003). Arranging these SST records into 69‐year timeseries for each location, we then calculated the mean (*x̄*_sst_) and coefficient of variance (cv_sst_) for each timeseries. Next, we estimated the frequency spectrum of each time series. Spectral analysis is used to quantify the periodicity of recurrent variability within a timeseries, with higher frequencies associated with shorter‐term fluctuations (Greenman & Benton, 2005). The frequency spectrum of a time series is represented by its spectral exponent (β) and equal to the slope between its log spectral density and log frequency (Gilljam et al., 2019), which we calculated using the package stats (R Core Team, 2019). After testing these abiotic predictor variables for collinearity (Appendix S1: Section S3), we performed our PLSR analyses using the R package plsdepot (Sanchez, 2012).

Finally, we assessed how patterns in the long‐term performance and capacity for coral assemblages to benefit from recurrent disturbance vary between tropical and subtropical regions, and how this variation manifests across coral taxa. Using a three‐way ANOVA, we separately investigated variation in our estimates of λ and maximal amplification across the three factors of country (Australia vs. Japan), ecoregion (tropical vs. subtropical), and assemblage classification (competitive, stress‐tolerant, or weedy). Prior to this analysis, data transformations applied to our estimates of maximal amplification (see *Quantifying population characteristics*) resulted in an inverted distribution for this variable. For the purposes of clarity, we henceforth refer to this reversed scale as a demographic stability index (DSI), whereby lower values correspond with an enhanced capacity for undergoing demographic amplification. We evaluated drivers of short‐ and long‐term performance, by using Type 2 linear regression to separately evaluate the relationship between generation time (*T*) and estimates of λ and TE. Type 2 linear regression is an approach for quantifying the relationship between two non‐independent variables, such that both variables include an element of error (Sokal & Rohlf, 1995). Here, due to differences in the magnitude of variance (σ^2^) across our variables of generation time, λ, and TE (σ^2^: *T* = 1.139; λ = 0.009; TE = 0.016) we performed a Ranged Major Axis Type 2 regression using the R package lmodel2 (Legendre, 2018).

## RESULTS

**FIGURE 2:**
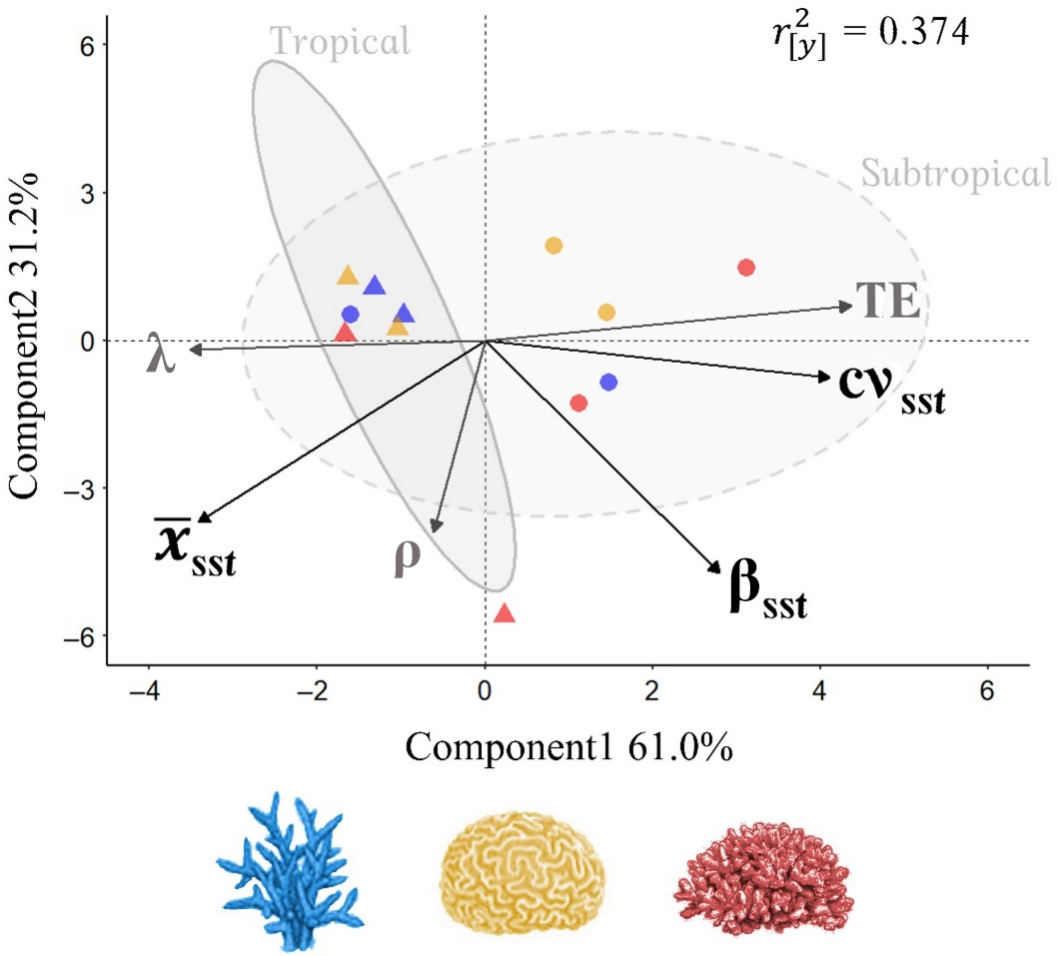
Contrasting patterns in long‐term performance and short‐term potential across our examined coral populations, corresponding with their relative exposure to abiotic variability. Partial least squares regression score plot illustrating the association between measures of abiotic variability, and the long‐term performance (λ) and short‐term potential (transient envelope [TE] and damping ratio [ρ]) of tropical (▲) and subtropical (●) populations of competitive (blue), stress‐tolerant (yellow), and weedy (red) coral taxa. We quantified the abiotic variability experienced by each coral population using representative measures of local sea surface temperature (SST) regimes (McIlroy et al., 2019; Toth et al., 2021). Specifically, we used SSTs recorded between 1950 and 2019 to calculate regional estimates of mean monthly SST (*x̄*_sst_), monthly SST variance (cv_sst_), and monthly SST frequency spectrum (β_sst_). Component scores illustrate the relative degree of variance explained in the abiotic predictor variables, whereas $r_{\left[y\right]}^{2}$ reflects the cumulative variance explained across the demographic characteristics. The shaded polygons reflect the clustering of tropical and subtropical populations, whereas the dotted lines delineate regions of association to facilitate the visualization of patterns in correlation between the abiotic and demographic variables.

We reveal contrasting patterns in long‐term performance and short‐term potential corresponding with the exposure of coral populations to abiotic variability, along a gradient from warmer, more stable environments to cooler, more variable conditions (Figure 2). Notably, coral assemblages exposed to more variable abiotic conditions display enhanced short‐term potential. Explaining 92.17% of the variance across our three measures of thermal exposure (*x̄*_sst_, cv_sst_, and β_sst_), our PLSR captures 37.43% of variance in our measures of long‐term performance (λ), demographic recovery (ρ), and short‐term potential (TE; Figure 2, $r_{\left[y\right]}^{2}$). The first PLSR component depicts a gradient in SST variability, describing 60.97% of the variance in thermal conditions experienced by our examined coral assemblages. It is along this component that divergent patterns within estimates of λ and TE are most pronounced. Consequently, estimates of TE correlate positively with the measures of thermal variability (cv_sst_) and frequency spectrum (β_sst_), while higher λ estimates are associated with warmer mean monthly SSTs (*x̄*_sst_; Figure 2). Meanwhile, damping ratio (ρ) estimates align with the second PLSR component that describes patterns in mean SST (*x̄*_sst_) and SST frequency (β_sst_).

**FIGURE 3:**
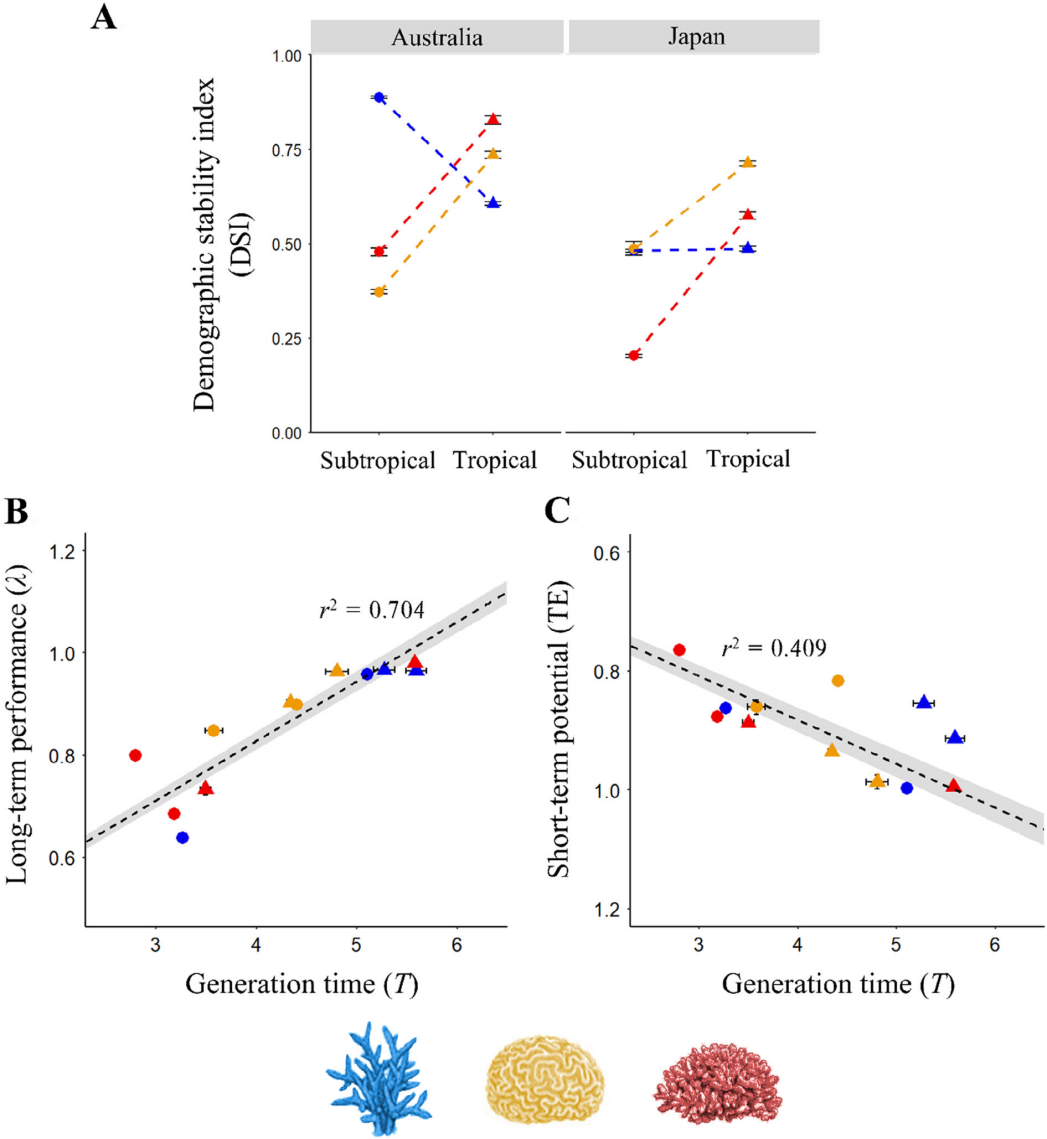
Inter‐specific variation within the contrasting patterns observed between long‐term performance and short‐term potential across tropical and subtropical coral assemblages correlates with patterns in population turnover rate. (A) Interaction plot showcasing how estimates of demographic stability index (DSI) vary between associated tropical (▲) and subtropical (●) assemblages of competitive (blue), stress‐tolerant (yellow), and weedy (red) coral taxa in Australia and Japan. We present DSI, as an inverse measure of maximal amplification (${\bar{\rho }}_{\max}$), describing the ability for populations to undergo elevated growth following disturbance. Thus, lower DSI estimates correspond with a greater capacity for demographic amplification. We also applied Type 2 linear regression to separately explore the association of population turnover characteristics with (B) long‐term performance (asymptotic population growth rate; λ), and (C) short‐term potential (transient envelope, TE) across tropical and subtropical populations of competitive, stress‐tolerant, and weedy coral taxa in Australia and Japan. We note here that TE estimates were reversed during transformation to achieve normality, thus higher values reflect diminished short‐term potential. We have therefore displayed short‐term potential on a reversed scale to facilitate comparisons with patterns in long‐term performance (λ). We used generation time (years; displayed here on the log scale) as a measure of population turnover rate, with higher estimates reflecting slower rates of population turnover. Across panels (B) and (C), *r* ^2^ values are provided as a measure of model fit. Across all panels error is displayed using 95% confidence intervals.

Although evident across taxa, the contrasting pattern we observe between long‐term performance and short‐term potential does not manifest consistently between paired tropical and subtropical coral assemblages (Figure 3A and Table 1). Our three‐way ANOVA highlighted significant interactions between the factors of assemblage classification (competitive, stress‐tolerant, or weedy), ecoregion (tropical vs. subtropical), and country (Australia vs. Japan; ANOVA_λ_: *F*_2,11,562_ = 5698.47, *p <* 0.001; ANOVA_DSI_: *F*_2,11,581_ = 589.8, *p <* 0.001). Despite this, the tropical assemblages routinely possess higher estimates of λ relative to their subtropical counterparts (Tukey: *p <* 0.001 in all cases; Table 1). The one exception was weedy corals in Japan, where λ is highest in the subtropics (λ_[*t*]_ = 0.760 [95% confidence intervals {CI}: 0.750, 0.770], λ_[*s*]_ = 0.807 [0.802, 0.812]; *p <* 0.001). Alternatively, our subtropical coral assemblages typically possess a greater capacity for undergoing demographic amplification following a disturbance than our tropical assemblages (Figure 3A). Yet, this pattern is not consistent across life‐history strategies, with competitive assemblages exhibiting the opposite trend in Australia (*p <* 0.001) and no variation in Japan (*p =* 0.999). Instead, the relative long‐term performance and short‐term potential of our examined tropical and subtropical coral assemblages correspond with patterns in their generation time (Figure 3B,C). Our Type 2 regression model shows generation time (*T*) to be a strong predictor of long‐term population growth rate (*r* ^2^ = 0.704), with long‐term performance increasing with generation time (Figure 3B). Conversely, longer generation times are associated with reduced short‐term potential (Figure 3C; *r* ^2^ = 0.409).

**TABLE 1:** Population growth rates (λ) obtained from corresponding tropical and subtropical assemblages of competitive, stress‐tolerant, and weedy coral taxa in Australia and Japan.

Country	Life‐history group	Tropical	Subtropical
Australia	Competitive	**0.983 [0.981, 0.984]**	0.958 [0.957, 0.959]
	Stress‐tolerant	**0.983 [0.980, 0.985]**	0.899 [0.898, 0.899]
	Weedy	**0.981 [0.980, 0.982]**	0.686 [0.684, 0.687]
Japan	Competitive	**1.001 [0.999, 1.004]**	0.640 [0.639, 0.641]
	Stress‐tolerant	**0.913 [0.909, 0.917]**	0.885 [0.877, 0.894]
	Weedy	0.760 [0.750, 0.770]	**0.807 [0.802, 0.812]**

*Note*: Bold text used to highlight the highest estimate of population growth across each tropical‐subtropical pairing. Error displayed using 95% confidence intervals.

## DISCUSSION

### Transient buffering in variable environments

Principally, contrasting patterns between long‐term performance and short‐term potential imply that long‐term performance does not predict the capacity for populations to endure repeated disturbances. Also, although enhanced short‐term potential may enable natural populations to persist within variable environments, it comes at a cost to their long‐term performance. Historically, variability in population growth rate was thought to diminish individual fitness (Pfister, 1998), thus hindering the persistence of populations (Lande, 1993). This understanding formed the basis of the demographic buffering hypothesis, whereby populations can minimize the influence of environmental stochasticity on their long‐term performance by limiting temporal variability in crucial vital rates (e.g., survival, development, and reproduction [Morris & Doak, 2004]). Thus, variable environments were assumed to select for populations with the ability to buffer key vital rates, thereby reducing temporal variation in performance characteristics (Hilde et al., 2020; Morris & Doak, 2004; Pfister, 1998). More recently, however, enhanced short‐term potential has been presented as an adaptive mechanism allowing populations to exploit more stochastic environments (McDonald et al., 2016). Ellis and Crone (2013) demonstrated how increased short‐term potential can buffer the effects of stochastic conditions on population growth rates, an effect that was increasingly evident in populations possessing lower λ estimates. Thus, it is not unexpected that coral assemblages established within variable environments would possess enhanced short‐term potential (Figure 2), but the energetic cost associated with this strategy would likely inhibit their long‐term performance characteristics.

Our finding that short‐term potential is greatest in coral assemblages displaying reduced long‐term performance contrasts with previous work on mammals and plants showcasing a positive association between population growth rates and short‐term potential (e.g., Gamelon et al., 2014; Morris et al., 2008). Higher population growth rates are assumed of populations characterized by faster individual development and high fecundity (Oli, 2004), with these populations also expected to exhibit greater variability in size following disturbances (Gamelon et al., 2014). While each of our surveyed assemblages are in, or close to, a state of long‐term decline (λ < 1; Table 1), projected long‐term performance was highest in the tropics, where relative capacities for demographic amplification were lowest (Figure 3A). Populations exhibiting longer generation times typically display reduced temporal variability in size due to higher investment in individual survival reducing the need to counteract disturbances (Morris et al., 2008); a pattern that we show to be evident in our examined coral assemblages (Figure 3C).

### Interspecific variation in short‐term potential

Variation in short‐term potential across our assemblages of differing coral taxa (Figure 3) suggests that exposure to abiotic variability alone does not assure resilience towards future climatic variability. Using data focused on a single taxon, Cant, Cook, et al. (2022) suggested that a capacity for short‐term increases in population growth observed in a subtropical *Acropora* spp. assemblage may underpin its viability in more variable high‐latitude environments. Here, we present evidence that this compensatory strategy is not just isolated to competitive coral taxa, but that stress‐tolerant and weedy coral taxa appear to possess a more pronounced capacity for demographic amplification at higher latitudes (Figure 3A). Weedy corals typically exhibit smaller colony sizes, faster growth rates, and brooding reproductive strategies, producing larvae that settle quickly after release (Darling et al., 2012; Knowlton, 2001). Together, these strategies support faster population turnover, enabling weedy coral species to proliferate within highly disturbed environments (Adjeroud et al., 2018). Conversely, stress‐tolerant corals display slower growth rates, longer life expectancies, high fecundity, and broadcast spawning strategies (Darling et al., 2012; Klepac & Barshis, 2020). The larger, more robust, morphologies associated with stress‐tolerant coral taxa maximize energy storage, promoting their persistence within challenging environments (van Woesik et al., 2012). Longer lifespans and elevated fecundity allow stress‐tolerant corals to endure stochastic conditions by taking advantage of sporadic improvements in local conditions (Darling et al., 2012). Consequently, our findings support existing projections that weedy and stress‐tolerant coral taxa are likely to become increasingly prevalent throughout disturbed coral assemblages (Cant et al., 2021; Loya et al., 2001). However, these projections herald the future loss of the structural complexity considered essential to the functioning of reef ecosystems (Graham & Nash, 2013).

We note that, while long‐term performance was typically highest in the tropics across each of our tropical‐subtropical assemblage pairings, the Japanese weedy coral assemblages show the opposite trend (Table 1). One possible explanation is that, in contrast to all other tropical‐subtropical assemblage pairings, the taxonomic composition of the Japanese weedy coral assemblages changed little between the tropics and subtropics (Appendix S1: Table S3). Consequently, we acknowledge that our observed patterns in the long‐term performance characteristics of each tropical and subtropical assemblage may result from their differing species compositions. However, it can also be argued, therefore, that the species compositions of subtropical coral assemblages allow them to exhibit an enhanced short‐term potential, relative to their tropical counterparts. Crucially, this scenario points to a potential driving mechanism responsible for the variation in species composition typically observed between tropical and subtropical coral assemblages. Abiotic stress generated by the more variable conditions associated with higher latitude environments selects for traits conferring a competitive advantage, filtering the species composition of subtropical coral assemblages (Sommer et al., 2018). From this perspective, our findings here support the environmental filtering hypothesis in subtropical coral assemblages, and present evidence that the selected traits relate to enhanced short‐term potential; a capacity that is not selected for in tropical environments.

Crucially, our findings here do not wholly reflect the current reality for many coral assemblages within regions of high abiotic variability, suggesting that the composition of coral assemblages is not solely mediated by the interplay between their short‐term dynamics and abiotic variability. Despite the reduced capacity for demographic amplification seen in subtropical competitive corals compared to subtropical weedy and stress‐tolerant populations, competitive coral taxa dominate many subtropical coral assemblages (Harriott et al., 1995; Nozawa et al., 2008; Sugihara et al., 2009). Utilizing fast growth strategies, colonies of competitive coral taxa are capable of rapidly colonizing available substrate, quickly outcompeting heterospecifics for both space and light (Darling et al., 2012). Whereas this competitive nature explains their dominance across contemporary subtropical communities, the sensitivity of many competitive coral taxa to environmental shifts means that these assemblages are often regarded as early successional, dominating only within optimal environments, and receding as reef ecosystems approach climax states (Ohba et al., 2008; Wilson et al., 2019). Within subtropical environments, coral community composition is mediated by environmental pressures and dispersal barriers that filter the occurrence of species according to their trait characteristics (Mizerek et al., 2021; Sommer et al., 2014). As a result, subtropical coral assemblages typically consist of a subset of tropical species found on tropical coral reefs (Sommer et al., 2017), as well as subtropical specialists and endemics. The dominance of competitive coral taxa within subtropical coral assemblages, despite their reduced short‐term potential relative to other coral taxa, may therefore imply that competitive interactions profoundly influence the performance of coral populations (Brito‐Millán et al., 2019; Idjadi & Karlson, 2007). Certainly, further investigation into the influence of competitive interactions upon the short‐term dynamics of coral populations is needed to disentangle how coexistence between coral populations facilitates their persistence within variable environments.

### Conclusions

A limited understanding for the abiotic determinants driving the dynamics of coral assemblages inhibits our capacity to predict their future performance and, therefore, manage global coral community reassembly (Edmunds, 2020; Edmunds et al., 2014; Edmunds & Riegl, 2020). Here, we demonstrate how coral populations can adopt demographic strategies associated with enhanced short‐term potential to improve their viability when exposed to greater abiotic variability. However, strategies of enhanced short‐term potential come at a cost to the long‐term demographic performance characteristics of coral populations. Despite presenting a framework for quantifying population resilience (Capdevila et al., 2020), the short‐term demographic characteristics of coral assemblages remain largely overlooked (Cant, Cook, et al., 2022). Yet, our findings emphasize that the winners and losers in coral assemblages exposed to more variable environments cannot be predicted using measures of long‐term performance. Moreover, our observed heterogeneity in the short‐term demographic characteristics of coral assemblages can help to explain why different assemblages display varying responses to periodic disturbances (Kim et al., 2023). This insight will benefit future predictions into the compositional reassembly of reef communities worldwide under future global change scenarios.

Consistent with documented shifts in the species composition of coral assemblages exposed to increased abiotic variability, our findings here highlight a key mechanism underlying the differential susceptibilities of coral species to periodic disturbance. Correlative assessments of community change over time illustrate how weedy and stress‐tolerant coral taxa in the Caribbean and tropical Atlantic have, in the past, fared better in periodically disturbed environments relative to competitive coral taxa (Cramer et al., 2021). Complementing these assessments, the variation we observed in the short‐term demographic potential of competitive, stress tolerant, and weedy coral taxa implies that enhanced short‐term demographic characteristics offer weedy and stress‐tolerant corals a greater capacity for enduring within frequently disturbed environments, relative to competitive coral species. With competitive coral taxa often considered paramount for supporting the structural complexity of coral reefs worldwide (Graham & Nash, 2013), this indictment compounds concerns for the future functioning and viability of global coral reef ecosystems. Crucially, by adopting a novel framework for quantifying demographic resilience, our work here contributes to disentangling the biotic and environmental drivers underpinning the diversity of coral responses to ongoing global change.

## AUTHOR CONTRIBUTIONS

James Cant, Roberto Salguero‐Gómez and Maria Beger developed the research ideas. James Cant, James D. Reimer, Brigitte Sommer, Katie M. Cook, Sun W. Kim, Carrie A. Sims, Takuma Mezaki, Cliodhna O'Flaherty, Hamish A. Malcolm, and Maria Beger collected field data, with James Cant, Cliodhna O'Flaherty, Maxime Brooks analyzing the data. James Cant, Roberto Salguero‐Gómez, and Maria Beger led the writing of the manuscript with all authors contributing critically to the writing and giving final approval for publication.

## CONFLICT OF INTEREST STATEMENT

The authors declare no conflicts of interest.

## Supporting information


Appendix S1.

## Data Availability

The raw demographic data used to parameterize the models developed for this manuscript are available on Dryad in Cant et al. (2023) at https://doi.org/10.5061/dryad.w0vt4b8xd. The R code needed to reproduce all the models and analyses presented is available from Zenodo (Cant et al., 2023) at https://doi.org/10.5281/zenodo.8059229.
